# Peer live supervision in the familial cancer setting

**DOI:** 10.1186/1897-4287-10-S2-A16

**Published:** 2012-04-12

**Authors:** M Kentwell, A Sexton, L Hodgkin, Y Bylstra, M Bogwitz, K Mann, J Taylor, M Sahhar, F Pearce, I Winship, GJ Lindeman

**Affiliations:** 1 Genetic Medicine and Familial Cancer, Royal Melbourne Hospital, Parkville, Victoria, Australia; 2 Victorian Clinical Genetics Services, Genetic Health, Parkville, Victoria, Australia

## 

At the Royal Melbourne Hospital, the Genetic Counsellors identified the desire to learn from each other, and engage in new forms of learning. Subsequently, a pilot project focusing on reflection, accountability, and experiential learning was developed. Live supervision with peers was identified as the model which fitted the learning objectives of the team.

The aims of the project were to:

1. Develop a live peer supervision model relevant to Genetic Counselling practice applicable to both Board Eligible and Certified Genetic Counsellors working in Familial Cancer.

2. Develop guided post observation questions based on reflective practice.

3. Identify key learning points from the live peer supervision sessions.

Seven Associate Genetic Counsellors/Genetic Counsellors participated in the project. The first phase involved collaborative brainstorming sessions to define the purpose of the project, gain knowledge and skills relevant to supervision, set the supervision contract, and develop post-observation questions.

After each live supervision session, the discussions of the observer and observee were audio recorded. The second phase of the project involved analysing the recorded discussions and identifying key areas of learning, relevant to clinical practice. The observer and observee's self-reported areas of learning were also identified. This presentation will describe the process and outcomes from these two phases of the project.

This pilot project provided the Genetic Counsellors with an opportunity to enhance clinical and supervision skills, harness the skills brought by each Genetic Counsellor, learn from one another's qualities, increase awareness of one's own style in clinical practice, and engage in the reflective process with immediacy.

There were a number of learning areas unique to this model of supervision, including access to experiential and visual learning.

Live-peer supervision may be a useful model to consider for both Associate and Certified Genetic Counsellors to engage in their continuing development and growth in clinical and supervision skills, ultimately improving patient care.

